# Rationale, design and population description of the CREDENCE study: cardiovascular risk equations for diabetes patients from New Zealand and Chinese electronic health records

**DOI:** 10.1007/s10654-021-00795-9

**Published:** 2021-08-22

**Authors:** Jingyuan Liang, Romana Pylypchuk, Xun Tang, Peng Shen, Xiaofei Liu, Yi Chen, Jing Tan, Jinguo Wu, Jingyi Zhang, Ping Lu, Hongbo Lin, Pei Gao, Rod Jackson

**Affiliations:** 1 Department of Epidemiology and Biostatistics, School of Public Health, Peking University, 38 Xueyuan Road, Beijing, 100191 China; 2 Section of Epidemiology and Biostatistics, School of Population Health, University of Auckland, 30 Park Avenue, Grafton, Auckland, 1023 New Zealand; 3 Yinzhou District Center for Disease Control and Prevention, Ningbo, China; 4 Peking University Clinical Research Institute, Peking University Health Science Center, Peking University, Beijing, China; 5 Chinese Evidence-Based Medicine Center, West China Hospital, Sichuan University, Chengdu, China; 6 Wonders Information Co. Ltd, Shanghai, China; 7 Key Laboratory of Molecular Cardiovascular (Peking University), Ministry of Education, Beijing, China

**Keywords:** Diabetes, Cardiovascular disease, Risk prediction, Electronic health records, Cohort

## Abstract

The cardiovascular risk equations for diabetes patients from New Zealand and Chinese electronic health records (CREDENCE) study is a unique prospectively designed investigation of cardiovascular risk in two large contemporary cohorts of people with type 2 diabetes from New Zealand (NZ) and China. The study was designed to derive equivalent cardiovascular risk prediction equations in a developed and a developing country, using the same epidemiological and statistical methodology. Two similar cohorts of people with type 2 diabetes were identified from large general population studies in China and New Zealand, which had been generated from longitudinal electronic health record systems. The CREDENCE study aims to determine whether cardiovascular risk prediction equations derived in patients with type 2 diabetes in a developed country are applicable in a developing country, and vice versa, by deriving and validating equivalent diabetes-specific cardiovascular risk prediction models from the two countries. Baseline data in CREDENCE was collected from October 2004 in New Zealand and from January 2010 in China. In the first stage of CREDENCE, a total of 93,207 patients (46,649 from NZ and 46,558 from China) were followed until December 31st 2018. Median follow-up was 7.0 years (New Zealand) and 5.7 years (China). There were 5926 (7.7% fatal) CVD events in the New Zealand cohort and 3650 (8.8% fatal) in the Chinese cohort. The research results have implications for policy makers, clinicians and the public and will facilitate personalised management of cardiovascular risk in people with type 2 diabetes worldwide.

**Supplementary Information:**

The online version contains supplementary material available at 10.1007/s10654-021-00795-9.

## Introduction

Diabetes is a major cause of cardiovascular disease (CVD) and risk prediction equations are increasingly used to identify diabetes patients at particularly high risk of CVD, to inform personalised treatment decisions [1]. A recent systematic review assessed the performance of 26 CVD risk prediction equations used in diabetes patients; 15 were derived in people with diabetes and 11 in general populations and later validated in people with diabetes [1]. The performance of equations in external validation studies was modest, at best, and many had not been externally validated. However, the ‘validity’ of many external validation studies of CVD risk prediction equations is questionable. These studies are frequently not ‘fair assessments’ as equations often have different inclusion and exclusion criteria and different outcome definitions from the populations they are ‘validated’ in, because validation populations are typically convenience samples from study populations recruited for other purposes [1]. Besides, developing countries have accounted for the majority of the global burden of diabetes, i.e. approximately 80% of people with diabetes are living in low- and middle-income countries as estimated by the International Diabetes Foundation [2]. However, among the currently available CVD risk prediction equations, very few were derived in developing countries [1]. Calibration of these equations has also not been assessed in these populations.

As far as we are aware, the **C**ardiovascular **R**isk **E**quations for **D**iabetes pati**E**nts from **N**ew Zealand and **C**hinese **E**lectronic health records (CREDENCE) study will be the first study to prospectively derive and validate comparable CVD risk prediction equations, for people with type 2 diabetes, simultaneously in a developed and developing country. This study will not only derive contemporary equations that can be applied to people with type 2 diabetes in New Zealand and China, but will also enable a fair comparison of equations derived in very different settings. If the equations perform well in both settings, this will also provide support for the current approach of deriving CVD risk prediction equations largely in developed countries and applying recalibrated versions in developing countries [3]. The present paper provides a detailed description of the CREDENCE study design, methodology and the study populations.

## Materials and methods

### Study design and participants

**Fig. 1:**
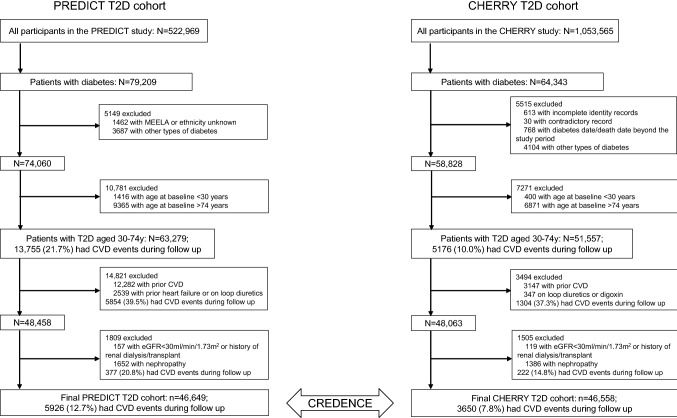
Design of the CREDENCE study: enrolment and incidence of first cardiovascular disease events. *MEELA* Middle Eastern, Latin American and African_,_ *eGFR* estimated glomerular filtration rate

The overall study design is shown in Fig. 1. The CREDENCE study consists of two diabetes cohorts from New Zealand and China respectively, i.e. the PREDICT-T2D cohort and the CHERRY-T2D cohort. The protocols of the general population PREDICT and CHERRY studies from which the diabetes patient cohorts were identified, have been previously described in detail [4, 5]. The two general population studies were both established using electronic health record (EHR) systems and have large sample sizes. In short, the PREDICT study was established in 2002 when a web-based CVD risk assessment and management decision support system (called “PREDICT”) was integrated into the EHR systems of approximately one-third of all New Zealand general practices. Participants are automatically recruited when they have a CVD risk assessment during a visit to a general practitioner, and by 2016 approximately 90% of all eligible patients had a completed CVD risk assessment. The CHERRY study is derived from the EHR system supporting the health care of Chinese residents living in Yinzhou District, Ningbo, Zhejiang Province aged over 18 years old. Approximately 98% of all Yinzhou District residents were registered in the EHR before 1st January, 2010.

### Inclusion and exclusion criteria

PREDICT is an open cohort, with participants entering following opportunistic risk assessments between 27th October, 2004 and 30th December, 2016. In the PREDICT study, some data items were entered onto the electronic PREDICT template within the EHR by the patient’s general practitioner at the time of the risk assessment, while other items were already in the EHR. The index date used in PREDICT was the date a patient’s CVD risk assessment was completed by their primary care practitioner, when all cardiovascular risk factors required for the risk assessment were available in the EHR. The PREDICT-T2D cohort included: (1) patients whose primary care EHR indicated they had type 2 diabetes at the time of their index CVD risk assessment; (2) patients dispensed oral hypoglycaemic agents or insulin at the time of assessment; or (3) those with diabetes-related ICD codes (Supplemental Table S1) in hospital discharge records prior to the index risk assessment. Required variables that were not already available in the EHR (e.g. duration of diabetes) were entered onto the PREDICT electronic template by the patient’s primary care practitioner at the time the risk assessment was completed.

**Fig. 2:**
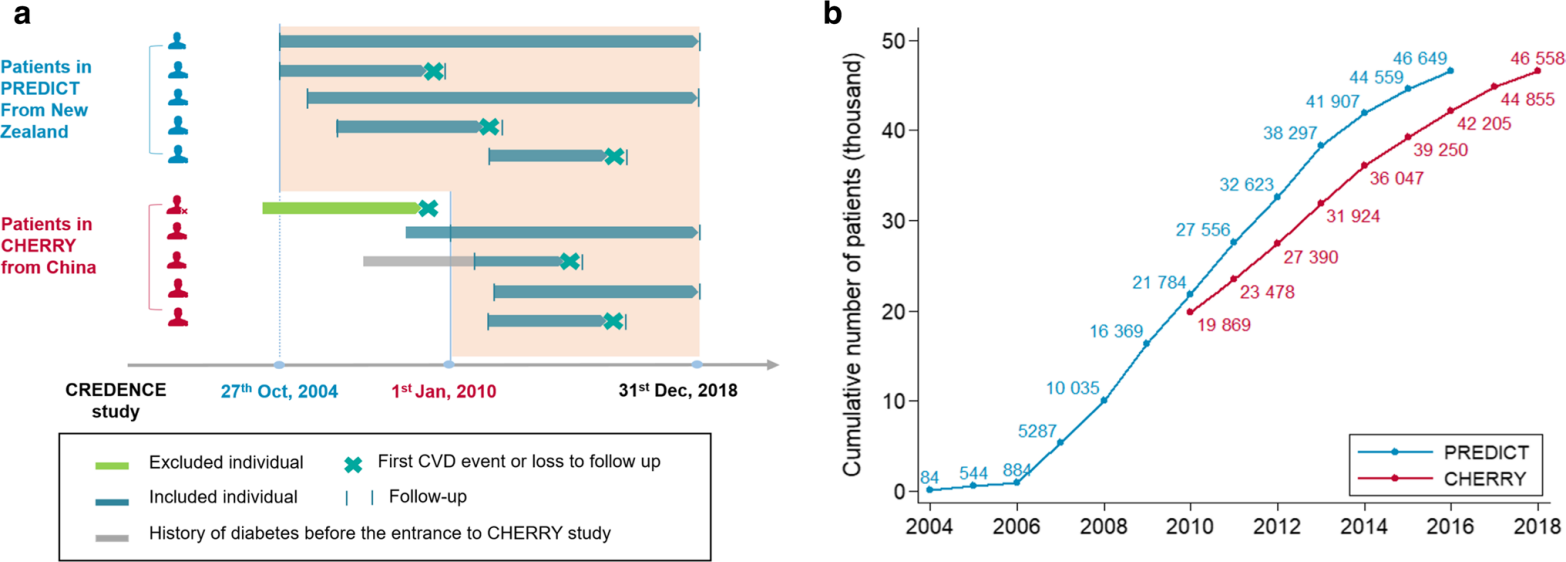
Participants enroled in the CREDENCE study. **a** Study design of the PREDICT T2D cohort and the CHERRY T2D cohort. In PREDICT, the index assessment date was the date participants were risk assessed, with the earliest date being 27th October, 2004 and the latest 30th December, 2016, and with follow-up to 31st December 2018. In CHERRY, patients were included if they (1) were in the original CHERRY cohort and were diagnosed with type 2 diabetes before 1st January 2010 (“previously diagnosed patients”), (2) entered the CHERRY cohort between 1st January 2010 and 31st December 2018 with history of diabetes (“previously diagnosed patients”), or (3) were newly diagnosed with diabetes while in the original CHERRY cohort during follow-up (“newly diagnosed patients”) between 1st January 2010 and 31st December 2018. **b** Numbers of participants recruited into the two CREDENCE study cohorts by visit year

In the CHERRY-T2D cohort, we included: (1) patients with type 2 diabetes who had already entered the CHERRY study at an index date set as 1st January 2010; (2) patients with diabetes who entered the CHERRY study after 1st January 2010, and their index date was set as their date of registration for health service; and (3) patients from the original CHERRY study who were newly diagnosed with type 2 diabetes between 1st January 2010 and 31st December 2018, where their index date was the date of diabetes diagnosis (Fig. 2). The duration of diabetes for patients in category (1) and (2) was defined as the difference between their index date and their date of diabetes diagnosis. For patients in category (3), it was coded as 0 at the index date. Diabetes status was obtained from three data sources with related ICD-10 codes (Supplemental Table S1) or equivalent Chinese text diagnoses: (1) diabetes-specific chronic disease management database; (2) diabetes surveillance system; or (3) inpatient electronic medical records database if patients had been discharged with the related ICD-10 code or Chinese text. For patients with multiple diagnosis dates available from different sources, the earliest one was selected as the date of diagnosis.

In the first stage of the CREDENCE study, we will derive CVD risk prediction equations for patients with diabetes. Patients from the two cohorts will be excluded if they:

1. are younger than 30 years or older than 74 years;
2. have a history of CVD, including heart failure;
3. have a history of renal dialysis, renal transplant, nephropathy or with estimated glomerular filtration rate (eGFR) lower than 30 ml/min/1.73 m^2^ at baseline.
4. have no valid healthcare identifier or had inconsistent or contradictory variables recorded across data sources.

History of prior CVD or renal dysfunction is defined by corresponding ICD-10 codes (Supplemental Table S1). Patients prescribed loop diuretics and digoxin (proxy for heart failure) will be also excluded.

We have applied identical ICD codes of relevant disease status used in the New Zealand and Chinese sites, selected after considering the differences in patients’ recruitment methods due to the different health care systems and disease screening programmes. After applying the same inclusion and exclusion criteria to the two studies, both PREDICT and CHERRY T2D cohorts included approximately 46,500 primary care patients with type 2 diabetes aged 30 to 74 years.

### Data definitions and measurements

Patient data are linked through various clinical and administrative databases. The healthcare information extracted can be grouped into 5 categories: sociodemographic data, primary care data, laboratory test results, pharmaceutical data and health outcomes. Measurements of individual patient's CVD risk factors were also available, with records from different sources merged through a unique identification number. Important risk factors for CVD risk prediction in patients with diabetes, that were common to both cohorts, included: age at index assessment, duration of diabetes, smoking status, body mass index, systolic blood pressure, eGFR (calculated using CKD-EPI equation [6]), urinary albumin, HbA_1c_, total and HDL cholesterol, history of atrial fibrillation, blood pressure lowering medication, lipid lowering medication and oral hypoglycaemic medication or insulin (Tables 1 and 2). The methods of measurement of risk factors in each of the cohorts are described in Supplemental Table S2.

**Table 1:** Characteristics of patients at index assessment in the CREDENCE study

Characteristics	PREDICT T2D cohort (n = 46,649)	CHERRY T2D cohort (n = 46,558)
*Sex*		
Male	23,994 (51.4%)	23,220 (49.9%)
Female	22,655 (48.6%)	23,338 (50.1%)
*Duration of diabetes, years*		
≤ 1	15,563 (33.4%)	32,306 (69.4%)
> 1	31,086 (66.6%)	14,252 (30.6%)
Age at index assessment, years	54.0 (11.0)	57.0 (9.6)
*Age group, years*		
< 35	1736 (3.7%)	711 (1.5%)
35–44	7805 (16.7%)	4898 (10.5%)
45–54	13,955 (29.9%)	12,920 (27.8%)
55–64	14,197 (30.4%)	17,752 (38.1%)
65–74	8956 (19.2%)	10,277 (22.1%)
*Ethnic group*		
European/Other	14,155 (30.3%)	0 (0.0%)
New Zealand Maori	7316 (15.7%)	0 (0.0%)
Pacific	11,807 (25.3%)	0 (0.0%)
Indian	7315 (15.7%)	0 (0.0%)
Chinese	3360 (7.2%)	46,558 (100.0%)
Other Asian	2696 (5.8%)	0 (0.0%)

Continuous data are presented as mean and standard deviation [mean (SD)], and categorical data are presented as number and percentage [n (%)]

**Table 2:** Medical history and index assessment measurement of CVD risk factors in CREDENCE study

Variable	PREDICT T2D cohort (n = 46,649)		CHERRY T2D cohort (n = 46,558)	
	Number measured	Summary statistics^a^	Number measured	Summary statistics^a^
Body mass index (kg/m^2^)	45,128	32.4 (7.5)	45,806	24.1 (3.1)
Systolic blood pressure (mmHg)	46,649	131.8 (15.4)	41,149	130.7 (12.0)
Diastolic blood pressure (mmHg)	46,648	80.3 (9.2)	41,158	80.1 (7.3)
Pulse Pressure (mmHg)	NA	NA	41,117	50.6 (9.9)
Smoking	46,648		34,690	
Current		7023 (15.1%)		6043 (17.4%)
Former		8761 (18.8%)		1331 (3.8%)
Never		30,864 (66.6%)		27,316 (78.7%)
*Glycemia*				
Fasting blood glucose (mmol/l)	NA	NA	40,188	7.2 (2.4)
Haemoglobin A1C (mmol/mol)	46,181	62.3 (20.7)	22,234	59.8 (21.8)
*Lipid profiles*				
Total cholesterol (mmol/l)	46,546	4.8 (1.1)	41,372	4.9 (1.1)
HDL cholesterol (mmol/l)	44,188	1.2 (0.3)	30,049	1.3 (0.4)
LDL cholesterol (mmol/l)	42,906	2.7 (0.9)	29,896	2.8 (0.9)
Non-HDL cholesterol (mmol/l)	44,186	3.6 (1.1)	30,007	3.6 (1.2)
Triglyceride (mmol/l)^b^	44,219	1.7 (1.2–2.4)	38,154	1.5 (1.1–2.2)
Total cholesterol: HDL ratio	46,536	4.2 (1.3)	30,025	3.9 (1.2)
*Renal function*				
eGFR (ml/min/1.73 m^2^)	40,914	89.6 (17.5)	38,785	95.6 (17.2)
Urinary albumin	NA	NA	34,481	
Normal				27,630 (80.1%)
Mild				5575 (16.2%)
Moderate				994 (2.9%)
Severe				282 (0.8%)
ACR (mg/mmol)^b^	41,510	1.4 (1.0–4.7)	10,178	2.0 (0.7–6.0)
*Medical history*				
Duration of diabetes, years	46,649	5.0 (5.5)	46,558	1.7 (3.5)
Atrial fibrillation	46,649	717 (1.5%)	46,558	22 (0.05%)
*Medications*				
Insulin	46,649	2947 (6.3%)	46,558	2314 (5.0%)
Oral hypoglycaemic agents	46,649	31,145 (66.8%)	46,558	25,967 (55.8%)
Blood pressure lowering medications	46,649	27,465 (58.9%)	46,558	24,798 (53.3%)
Lipid lowering medications	46,649	25,321 (54.3%)	46,558	8806 (18.9%)
Statin	46,649	23,109 (49.5%)	46,558	8126 (17.5%)

*NA* not available, *HDL* high density lipoprotein, *LDL* low density lipoprotein, *eGFR* estimated glomerular filtration rate, *ACR* albumin to creatinine ratio

^a^Continuous data are presented as mean and standard deviation [mean (SD)] unless otherwise stated, and categorical data are presented as number and percentage [n (%)]

^b^presented as median and interquartile range (IQR) [median (IQR)]

### Outcomes

For each patient, data was sought on each of the following outcomes and their dates of diagnosis: non-fatal CHD, non-fatal stroke, transient ischaemic attacks (TIA), cause-specific CVD mortality (e.g., fatal CHD and fatal stroke) and other cardiovascular diseases. The primary outcome will be CVD defined according to the ICD-10 codes listed in Table 3. Secondary outcomes will include fatal and non-fatal CHD and stroke (Table 3). The PREDICT study outcomes are all identified from national hospitalisation and mortality collections, whereas clinical outcomes in the CHERRY study are obtained by linking the participants to relevant databases (the death/disease surveillance database, the chronic disease management database, and inpatient EMR databases) within the Yinzhou integrated health information system, by a unique and encoded identifier.

**Table 3:** Definitions of cardiovascular outcomes in the CREDENCE study

Outcome	Events of interest	ICD-10 codes	
		Non-fatal	Fatal
*Primary events of interest*			
CVD	Coronary heart disease	I20-I25, I46 (excluding I461)	I20-I25, I46
	Heart failure	I50 (excluding I508), I110, I130, I132	I50 (excluding I508), I110, I130, I132
	Stroke	I60, I61, I63, I64	I60, I61, I63, I64
	Transient ischaemic attacks	G45 (excluding G454)	G45 (excluding G454)
	Other cerebrovascular diseases	I66, I670	I66, I67, I672, I69 (excluding I692)
	Peripheral vascular disease	I65, I702, I710, I711, I713, I715, I718, I739, I74	I65, I70, I71, I739, I74
*Secondary events of interest*			
CHD	Angina pectoris	I20	I20
	myocardial infarction,	I21-I23	I21-I23
	other Chronic ischemic heart disease	I24, I252-I257	I24, I25
	cardiac arrest	I46(excluding I461)	I46
Stroke	Haemorrhagic stroke	I60, I61	I60, I61
	Ischaemic stroke	I63, I64	I63, I64
	Transient ischaemic attacks	G45 (excluding G454)	G45 (excluding G454)

Definitions are slightly different between fatal and non-fatal CVD considering the specific meanings of codes and the practical application situation in New Zealand and China

*CVD* Cardiovascular disease, *CHD* Coronary heart disease

### Follow-up

Both PREDICT and CHERRY studies were designed with electronic follow-up procedures. In the PREDICT study, primary care patients are “electronically followed” every 1–2 years through encrypted National Health Index (NHI) number linkage to routine national hospitalisation and mortality databases. In CHERRY, records of CVD, other chronic diseases and deaths are updated annually. In the first stage of CREDENCE, the follow-up time was defined from index assessment (starting from 27th October, 2004 in the PREDICT T2D cohort and 1st January, 2010 in the CHERRY T2D cohort) until the first CVD event, death due to other causes, withdrawal from the cohort for various reasons or end of follow-up (31st December, 2018 in both studies). We plan to extend the follow-up period in future study stages.

### Statistical approach

We will follow the Transparent Reporting of a multivariable prediction model for Individual Prognosis Or Diagnosis (TRIPOD) guidelines [7]. This first stage of CREDENCE will involve the development of 5-year CVD risk prediction equations using Cox regression models in each of the study cohorts. Details of the risk prediction equations will be published to enable readers to develop CVD risk calculators, and will include all regression coefficients and baseline survival at 5 years. Validation of the prediction models will include internal validation (bootstrapping or cross-validation) as well as external validation using the counterpart’s participants. Key aspects for the assessment of model performance will include discrimination, calibration, reclassification and explained variation. To assess clinical utility among various risk thresholds for intervention, decision curve analysis will also be performed [8]. Standard recalibration methods will also be used to ensure better agreement between the model-predicted and observed events in the two cohorts. Moreover, to facilitate implementation of the models in busy routine clinical practice, simple CVD risk prediction models will be simultaneously established alongside the full models.

We have presented the proportions of missing values for each proposed risk predictors in the two CREDENCE cohorts in Table 4. To avoid excluding observations with missing data, which may produce biased results, multiple imputation by chained equations [9] will be used to impute missing values for the predictors with incomplete data. All statistical analyses in this report were carried out using SAS, V9.4 (SAS Institute, Cary, North Carolina, USA) and Stata software, V15.1 (StataCorp, College Station, Texas, USA).

**Table 4:** Missing proportions of predictors in CREDENCE study

Predictors	PREDICT T2D cohort n = 46,649	CHERRY T2D cohort n = 46,558
Body mass index (kg/m^2^)	1521 (3.3%)	752 (1.6%)
Systolic blood pressure (mmHg)	0 (0.0%)	5409 (11.6%)
Diastolic blood pressure (mmHg)	1 (< 0.1%)	5400 (11.6%)
Pulse Pressure (mmHg)	NA	5441 (11.7%)
Smoking	1 (< 0.1%)	11,868 (25.5%)
*Glycemia*		
FBG (mmol/l)	NA	6370 (13.7%)
HbA_1C_ (mmol/mol)	468 (1.0%)	24,324 (52.2%)
*Lipid profiles*		
Total cholesterol (mmol/l)	103 (0.2%)	5186 (11.1%)
Triglyceride (mmol/l)	2430 (5.2%)	8404 (18.1%)
Total cholesterol: HDL ratio	113 (0.2%)	16,533 (35.5%)
*Renal function*		
eGFR (ml/min/1.73 m^2^)	5735 (12.3%)	7773 (16.7%)
Urinary albumin	NA	12,077 (25.9%)
ACR (mg/mmol)	5139 (11.0%)	36,380 (78.1%)

Categorical data are presented as number and percentage [n (%)]

*NA* not available, *FBG* fasting blood glucose, *HbA*_*1c*_ haemoglobin A1C, *HDL* high density lipoprotein, *eGFR* estimated glomerular filtration rate, *ACR* albumin to creatinine ratio

### Data management and privacy protection

In PREDICT and CHERRY, all data are linked using unique encrypted identifiers. Sets of logical, technical and administrative controls are implemented. Data access rights are assigned to personnel according to their role in the study. Third-party companies (Enigma Solutions Ltd for PREDICT and Wonders Information for CHERRY) are engaged, where necessary, to handle data extraction, linkage and storage to ensure privacy protection. Currently, data contained in the CREDENCE study are not freely available on a public server. Therefore, scientists who are interested in the CREDENCE study cohorts should contact the study investigators regarding potential collaborations.

### Results

As shown in Fig. 1, in the first stage of CREDENCE, there were a total of 93,207 patients with diabetes in the two cohorts, including 46,649 from PREDICT and 46,558 from CHERRY T2D cohorts. Almost 95% of all patients with diabetes in the PREDICT T2D cohort were recorded by their general practitioner as having diabetes on the PREDICT template, and in the CHERRY T2D cohort, 90% had been recorded as having diabetes in at least two sources (i.e. diabetes-specific chronic disease management database, diabetes surveillance system and electronic medical records).

Demographic characteristics of the CREDENCE cohorts, stratified by country and sex, are presented in Table 1. The overall mean age at index assessment was 54.0 (standard deviation (SD) 11.0) years for patients from New Zealand and 57.0 (9.6) years for patients from China. 22,655 (48.6%) patients in PREDICT-T2D and 23,338 (50.1%) in CHERRY-T2D were women. Of note, 7.2% (3360) of the patients in the PREDICT-T2D cohort are descendants of Chinese immigrants or recent Chinese migrants, which will allow us to compare the accuracy of risk prediction models derived respectively from the current PREDICT and CHERRY populations, specifically applied to Chinese people in each study.

Medical history and baseline measurement of CVD risk factors in the two CREDENCE cohorts are presented in Table 2 and Supplemental Table S3. The mean duration of diagnosed diabetes was 5 years in PREDICT-T2D, but only 1.7 years in CHERRY-T2D. A total of 14,252 (30.6%) patients had over a 1-year history of diabetes at baseline in the CHERRY-T2D cohort, whereas 31,086 (66.6%) patients in the PREDICT cohort have been living with diabetes for over a year. Patients with diabetes in PREDICT had a much higher mean BMI compared with patients in CHERRY (32.4 kg/m^2^ and 24.1 km/m^2^, respectively). The New Zealand study participants also had, on average, higher levels of HbA_1c_ (62.3 mmol/mol in PREDICT-T2D and 59.8 mmol/mol in CHERRY-T2D), and lower levels of eGFR (89.6 ml/min/1.73m^2^ in PREDICT-T2D and 95.6 ml/min/1.73m^2^ in CHERRY-T2D). The prevalence of current smoking was twice as high among Chinese men (36%) versus New Zealand men (17%), whereas fewer than 1% of Chinese women were current smokers, compared with more than 13% of New Zealand women (Supplemental Table S3). Proportions of patients on several medications, particularly statins, were also higher in the PREDICT-T2D cohort, and more patients in the New Zealand cohort had atrial fibrillation at index assessment. Other predictors are broadly comparable in the two cohorts.

Table 5 summarises the follow-up time and occurrence of CVD events among patients in the CREDENCE cohorts. There were a total 9576 CVD events in this first stage of CREDENCE, with New Zealand patients experiencing 5926 (61.9%) of these. Proportions of CVD subtypes varied across the two cohorts. In CHERRY-T2D, 2221 patients had a stroke during follow-up, accounting for 60.8% of total CVD events, whereas stroke accounted for only 29% of PREDICT-T2D CVD events.

**Table 5:** Follow-up time and occurrence of CVD events among patients in CREDENCE study

Endpoint	Total			Men			Women		
	N with events, %		Rate, per 1000 Person-years	N with events, %		Rate, per 1000 Person-years	N with events, %		Rate, per 1000 Person-years
*PREDICT T2D cohort*	Median follow-up: 7.0 years			Median follow-up: 6.9 years			Median follow-up: 7.1 years		
All CVD	5926 (12.7%)	18.3 (17.8, 18.7)		3547 (14.8%)	21.4 (20.7, 22.1)		2379 (10.5%)	15.0 (14.4, 15.6)	
Fatal CVD	454 (1.0%)	1.3 (1.2, 1.4)		283 (1.2%)	1.6 (1.4, 1.8)		171 (0.8%)	1.0 (0.9, 1.2)	
Non-fatal stroke	1604 (3.4%)	4.7 (4.5, 5.0)		874 (3.6%)	5.0 (4.7, 5.3)		730 (3.2%)	4.4 (4.1, 4.8)	
Fatal stroke	116 (0.3%)	0.3 (0.3, 0.4)		50 (0.2%)	0.3 (0.2, 0.4)		66 (0.3%)	0.4 (0.3, 0.5)	
Non-fatal CHD	2693 (5.8%)	8.0 (7.7, 8.3)		1671 (7.0%)	9.7 (9.3, 10.2)		1022 (4.5%)	6.3 (5.9, 6.6)	
Fatal CHD	293 (0.6%)	0.8 (0.8, 1.0)		207 (0.9%)	1.2 (1.0, 1.3)		86 (0.4%)	0.5 (0.4, 0.6)	
*CHERRY T2D cohort*	Median Follow-up: 5.7 years			Median follow-up: 5.5 years			Median follow-up: 6.0 years		
All CVD	3650 (7.8%)	13.5 (13.0, 13.9)		1840 (7.9%)	14.0 (13.4, 14.7)		1810 (7.8%)	12.9 (12.4, 13.6)	
Fatal CVD	323 (0.7%)	1.2 (1.1, 1.3)		159 (0.7%)	1.2 (1.0, 1.4)		164 (0.7%)	1.2 (1.0, 1.4)	
Non-fatal stroke	2036 (4.4%)	7.5 (7.2, 7.8)		1029 (4.4%)	7.8 (7.4, 8.3)		1007 (4.3%)	7.2 (6.8, 7.7)	
Fatal stroke	185 (0.4%)	0.7 (0.6, 0.8)		93 (0.4%)	0.7 (0.6, 0.9)		92 (0.4%)	0.7 (0.5, 0.8)	
Non-fatal CHD	245 (0.5%)	0.9 (0.8, 1.0)		163 (0.7%)	1.2 (1.1, 1.4)		82 (0.4%)	0.6 (0.5, 0.7)	
Fatal CHD	45 (0.1%)	0.2 (0.1, 0.2)		20 (0.1%)	0.2 (0.1, 0.2)		25 (0.1%)	0.2 (0.1, 0.3)	

*CVD* cardiovascular disease, *CHD* coronary heart disease

## Discussion

The CREDENCE study is a unique international comparative study in which patients with type 2 diabetes, meeting specified inclusion and exclusion criteria, have been prospectively identified from EHR-based general population cohort studies in New Zealand and China. In the first stage of the study, we aim to derive and evaluate CVD risk prediction models using the same methodological framework.

The prevalence of diabetes is increasing in most countries. CVD risk assessment of patients with diabetes is essential for effective personalised disease management [10–12]. Numerous risk prediction models have been developed to identify patients with diabetes at high risk of CVD. These models can be classified into two categories: those developed using a general population with a binary diabetes status indicator (e.g. Framingham risk score [13] from US, QRISK [14] from UK and PREDICT [15] from New Zealand), and others derived using only patients with diabetes (e.g. U.K. Prospective Diabetes Study model [16], ADVANCE model [17] from 20 countries, NZDCS equation and PREDICT-1° Diabetes equation [18, 19] from New Zealand and DIAL algorithm [20] from Sweden).

Though models in the first category are simple to implement in clinical practice, many key predictors for patients with diabetes are not included, e.g. diabetes duration, renal function, diabetes pharmacotherapies. A number of attempts have been made to compare the performance of diabetes-specific models versus general population models, with diabetes-specific models generally performing better than general population models [21]. However, few diabetes-specific models have been derived in developing countries, including China, and the performance of existing diabetes-specific models has not been assessed in diverse populations. Moreover, many prediction models are derived from cohorts recruited decades ago when CVD risk factor distributions and event rates were very different from today, in both developing and developed countries.

Given these issues, it is unknown which CVD risk prediction models will perform best in different populations. Model comparisons and validation studies have also been complicated by between-study variation in inclusion criteria, study timeframes, predictor and outcome definitions, distributions of risk factors, duration of follow-up, as well as differences in background risks of study populations. As a result, few comparative studies can be considered fair comparisons. The CREDENCE study aims to address these issues by adopting a uniform approach to development and validation of risk prediction models, derived in two cohorts from very different settings, that were prospectively defined to ensure they were comparable.

### Strengths and limitations

The CREDENCE study has a number of strengths. Firstly, the study is a prospectively designed comparative study conducted in New Zealand and Chinese type 2 diabetes patient cohorts, in which equivalent CVD risk prediction equations will be developed. The PREDICT study in New Zealand and the CHERRY study in China, from which the diabetes cohorts were identified, are comparable large-scale population-based cohort studies derived from EHR systems. Although some baseline characteristics of patients with diabetes are different in the two cohorts, that is to be expected and we have applied the same inclusion and exclusion criteria for cohort entry to minimise these differences. Furthermore, we have used comparable definitions of risk predictors and outcomes and we will develop and evaluate CVD risk prediction models using the same methodological framework, including consistent methods of modelling and performance assessment, etc. This also allows the prediction models developed in one cohort to be externally validated in the other, hence providing information on generalisability of models.

Secondly, the study can provide contemporary CVD risk prediction equations for use in patients with type 2 diabetes in New Zealand and China, to fill an important research and practice gap. To the best of our knowledge, no diabetes-specific CVD risk prediction model has been developed in mainland Chinese patients with type 2 diabetes in a real-world population. Finally, both cohorts are large, natural population-based cohorts with repeated longitudinal measurements specifically designed to investigate CVD risk management and clinical outcomes. This will facilitate research on how risk factors influence the onset and progression of CVD events in patients with diabetes over the life course.

Routinely collected electronic health data has some advantages over data collected in epidemiological research studies in terms of size, generalisability and representativeness, as well as completeness of follow-up when it can be linked to comprehensive outcome databases. According to the TRIPOD guidelines, prediction models using real world data such as data from healthcare records are recommended for both public health and clinical practice. However, this approach generally involves less comprehensive and less precise protocols for data collection compared to traditional research studies.

The study also has several other limitations. Firstly, both original cohorts draw information from administrative databases and electronic health records that are designed for healthcare management but not for the epidemiological research. Therefore, there could be possible misclassification regarding the subtypes of diabetes. However, this misclassification is likely to be very small as the incidence of type 2 diabetes is much higher than type 1 diabetes, and the age of diagnosis was over 30 years for most patients (95.7% in PREDICT-T2D and 99.7% in the CHERRY-T2D); most type 1 diabetes is diagnosed at much younger ages [2]. Secondly, although the PREDICT study incorporated a prospectively designed risk prediction template as an integrated clinical and research tool, there were still missing values for some variables in the administrative databases. Missing values were more common in CHERRY, as all variables came from administrative databases. Finally, although the CHERRY study has a relatively large number of participants, it is a regional cohort located in a developed area of China and as such, will not be nationally representative.

## Conclusion

The CREDENCE study is an international collaboration in which equivalent CVD risk prediction equations will be derived from comparable cohorts of patients with type 2 diabetes: one from New Zealand and the other from China. The cross-country nature of the study design, the use of comparable analytical approaches, coverage of the total diabetes populations within defined geographic regions, and the availability of repeated measurements of cardiovascular risk factors and clinical outcomes, make it a unique resource for research on the CVD risk and risk management.

## Supplementary Information

Below is the link to the electronic supplementary material.

Supplementary file 1 (DOCX 42 kb)

## Data Availability

Not applicable.
