# Supplementary material for: Rationale, design and population description of the CREDENCE study: cardiovascular risk equations for diabetes patients from New Zealand and Chinese electronic health records
Source: Eur J Epidemiol. 2021 Aug 22;36(10):1085–95. doi: 10.1007/s10654-021-00795-9 (PMC8542537; doi:10.1007/s10654-021-00795-9)
Supplement: Supplementary file 1 — (DOCX 42 kb) [file 10654_2021_795_MOESM1_ESM.docx]

**Supplementary Material**

**Rationale, Design and Population Description of the CREDENCE Study: Cardiovascular Risk Equations for Diabetes patiEnts from New Zealand and Chinese Electronic health records**

**Short Title: The CREDENCE Study**

**Journal: European Journal of Epidemiology**

Jingyuan Liang^1#^, Romana Pylypchuk^2#^, Xun Tang^1^, Peng Shen^3^, Xiaofei Liu^1,4^, Yi Chen^1^, Jing Tan^5^, Jinguo Wu^6^, Jingyi Zhang^6^, Ping Lu^6^, Hongbo Lin^3^, Pei Gao^1,4,7^*, Rod Jackson^2^*

1. Department of Epidemiology and Biostatistics, School of Public Health, Peking University, Beijing, China
2. Section of Epidemiology and Biostatistics, School of Population Health, University of Auckland, Auckland, New Zealand
3. Yinzhou District Center for Disease Control and Prevention, Ningbo, China
4. Peking University Clinical Research Institute, Peking University Health Science Center, Peking University, Beijing, China
5. Chinese Evidence-based Medicine Center, West China Hospital, Sichuan University, Chengdu, China
6. Wonders Information Co.Ltd, Shanghai, China
7. Key Laboratory of Molecular Cardiovascular (Peking University), Ministry of Education, Beijing, China

^#^ Equal contribution as the first author; * Equal contribution as the corresponding author.

**Correspondence:**

Prof. Pei Gao ([peigao@bjmu.edu.cn](mailto:peigao@bjmu.edu.cn)): 38 Xueyuan Road, Beijing 100191, China. Tel/Fax: +86-10-82805642; or Prof. Rod Jackson (rt.jackson@auckland.ac.nz): 30 Park Avenue, Grafton, Auckland 1023, New Zealand. Tel/Fax: +64-99236343.

**Supplemental Table S1.** Definitions of diabetes and prior diseases in the CREDENCE study

| **Diseases** | **ICD-10** |
| --- | --- |
| **Diabetes** |  |
| Non-insulin-dependent diabetes mellitus | E11 |
| Other specified diabetes mellitus | E13 |
| Unspecified diabetes mellitus | E14 |
| **Prior CVD** |  |
| CVD | G45, I11.0, I13.0, I13.2, I20-I25, I46 (excluding I461), I50 (excluding I50.8), I60, I61, I63-I66, I67.0, I67.2, I69 (excluding I69.2), I70, I71, I73.9, I74 |
| Transient ischaemic attacks | G45 |
| Heart failure | I11.0, I13.0, I13.2, I50 (excluding I50.8) |
| Coronary heart disease | I20-I25, I46 (excluding I461) |
| Stroke | I60, I61, I63, I64 |
| Peripheral vascular disease | I65, I70.0, I71, I73.9, I74 |
| Other cerebrovascular diseases | I66, I67.0, I67.2, I69 (excluding I692) |
| **Prior renal dysfunction** | |
| Nephropathy | N00-N19, E10.2, E11.2, E12.2, E13.2, E14.2 |
| Renal transplant | T86.1, Z94.0 |
| Renal dialysis | T82.4, T85.7, Y60.2, Y61.2, Y62.2, Y84.1, Z45.2, Z49, Z99.2 |
| **Atrial Fibrillation** | I48 |

Abbreviations: CVD: cardiovascular disease.

**Supplemental Table S2.** Risk predictors to be included in sex-specific CREDENCE models and their methods of measurement

| **Predictors** | **Measurement methods** | |
| --- | --- | --- |
|  | **PREDICT T2D** | **CHERRY T2D** |
| Age at baseline in years, continuous | This variable was derived from the participant’s index assessment date and their date of birth. Date of birth is a component of the National Health Index (NHI) dataset on all New Zealanders and was automatically linked to the PREDICT dataset. | Patients’ ID numbers were derived from population census and registered health insurance database and their date of birth was then identified (which is recorded in this number as eight digits) |
| Duration of diabetes in years, continuous | This variable was calculated as the difference between the age at baseline and the age when diagnosed as diabetes. | |
| Body mass index in kg/m^2^, continuous | BMI was calculated as weight(kg)/height(m)^2^. Weight and height recorded on the same measurement date and in the same database were used in calculation. These were the most recently recorded weight and height levels at the time of the index assessment. | |
| Blood pressure in mmHg, continuous | These were the mean of the two most recently recorded sitting blood pressure levels at the time of the index assessment, measured by either a general practitioner or practice nurse. | This was measured by either a general practitioner (population census and registered health insurance database, disease manage database) or practice nurses (health checks database). |
| Smoking status, categorical | This variable was recorded by the health professional completing the electronic form. The categories provided were: no-never; no – quit over 12 months ago; no – quit less than 12 months ago; yes – up to 10/day; yes – 11-19/day; yes – 20+/day. These were combined into three categories (non-smoker, ex-smoker, current smoker) based on preliminary risk models. | Smoking status of patients were recorded in several databases, mainly including health checks database and population census and registered health insurance database. They were recorded as categorical variables (non-smoker, ex-smoker, current smoker) or continuous variables (number of cigarettes smoked per day) due to different design forms. These were combined into three categories based on preliminary risk models. |
| Glycemia, continuous | The HbA_1c_ (mmol/mol) measurement was the most recent result recorded before index risk assessment, or, if none were available, up to 14 days after baseline. If a patient didn’t have at least one result in this period, but was not on blood glucose-lowering drugs (insulin or oral hypoglycaemic), an HbA_1c_ measurement was included up to 18 months post index assessment. | The HbA_1c_ (mmol/mol) and FBG (mmol/l) were recorded in disease management database, health checks database and inpatient EMR. |
| Lipid profiles, continuous | These were the most recently recorded lipid levels at the time of the index assessment. They are measured in community laboratories and are automatically downloaded into patient records. | The lipid profiles were measured in community laboratories or hospitals. Total cholesterol and HDL levels recorded in the same measurement date and the same database were used in the calculation of total cholesterol:HDL ratio. |
| eGFR in ml/(min·1.73m^2^)^-1^, continuous | The eGFR was calculated using the Chronic Kidney Disease Epidemiology Collaboration (CKD-EPI) equation as recommended by the 2013 guidelines from the Kidney Disease: Improving Global Outcomes Organization. Serum creatinine used in the calculation was based on the most recent result recorded before the index risk assessment, or, if none were available, up to 12 months after the index assessment. | The eGFR was calculated using the CKD-EPI equation. Serum creatinine level used in the equation was recorded in health checks database and inpatient EMR. |
| Urinary albumin, categorical | NA | This variable was categorised as: normal (with negative result), mild (a plus sign), moderate (two plus signs) or severe (no less than two plus signs). The measure was recorded in disease management database, health checks database and inpatient EMR. |
| ACR in mg/mmol, continuous | ACR was based on the most recent laboratory results recorded before the index risk assessment, or, if none available, up to 12 months after the index assessment. | ACR was recorded in health checks database and inpatient EMR. |
| History of atrial fibrillation, categorical | ECG confirmed AF was recorded by the health professional completing the electronic form. In addition, if participants had been hospitalised with atrial fibrillation they were classified as having atrial fibrillation. | This variable was identified using related ICD-10 code (EMRs) or Chinese characters (population census and registered health insurance database and disease management database). |
| Blood pressure lowering medication, categorical | Blood pressure lowering medication (including angiotensin converting enzyme (ACE) inhibitors, beta-blockers, thiazide, angiotensin II receptor blockers (ARB) and calcium channel blockers) dispensed during the six months prior to the index risk assessment. This variable was extracted from the National drug dispensing database and primary care records. | Categories of blood pressure lowering medication were the same as for PREDICT plus alpha-blockers. If participants were prescribed blood pressure lowering medication prior to the study index assessment, they were classified as having blood pressure lowering medication. The information was extracted from disease management database, health checks database and inpatient EMR. |
| Lipid lowering medication, categorical | Lipid-lowering medication (including atorvastatin, fluvastatin, pravastatin, simvastatin, acipimox, bezafibrate, cholestyramine, clofibrate, colestipol, ezetimibe, ezetimibe with simvastatin, gemfibrozil and nicotinic acid) dispensed during the six months prior to the index risk assessment. This variable was extracted from the National drug dispensing database and primary care records. | Lipid-lowering medication includes statins, nicotinic acid, cholesterol absorption inhibitors, probucol, cholic acid chelating agent, fibrates. If participants were prescribed lipid lowering medication prior to the study index assessment, they were classified as having lipid lowering medication. The information was extracted from disease management database, health checks database and inpatient EMR. |
| Hypoglycaemic medication and insulin, categorical | Oral hypoglycaemic agents (including acarbose, chlorpropramide, glibenclamide, gliclazide, glipizide, metformin, pioglitazone, rosiglitazone, tolazamide and tolbutamide), dispensed at least once during six months prior to the index risk assessment. All subsidised forms of insulin dispensed at least once during six months prior to the index risk. This variable was extracted from National drug dispensing database and primary care records. | Oral hypoglycaemic agents include biguanides, sulfonylureas, non-sulfonylurea derivatives of anisic acid, alpha-glucosidase inhibitors, thiazolidinediones, glucagon-like peptide 1 (GLP-1) receptor agonist, dipeptidyl peptidase IV (DPP-4) inhibitors and sodium glucose transporter 2 (SGLT2) inhibitors. If participants were prescribed hypoglycaemic medication or insulin before the study index assessment, they were classified as having lipid lowering medication or insulin. The information was extracted from disease management database, health checks database and inpatient EMR. |

Abbreviations: NA: not available; HDL: high density lipoprotein; eGFR: estimated glomerular filtration rate; ACR: albumin to creatinine ratio;

FBG: fasting blood glucose; HbA_1c_: haemoglobin A1C

**Supplemental Table S3.** Medical history and first measurement of CVD risk factors in CREDENCE study, by sex

| **Variable** | **PREDICT T2D cohort** | | | **CHERRY T2D cohort** | | |
| --- | --- | --- | --- | --- | --- | --- |
|  | **Total,**  **n=46,649** | **Male,**  **n=23,994** | **Female,**  **n=22,655** | **Total,**  **n=46,558** | **Male,**  **n=23,220** | **Female,**  **n=23,338** |
| Body mass index (kg/m^2^) | 32.4 (7.5) | 31.4 (6.8) | 33.5 (8.1) | 24.1 (3.1) | 24.1 (2.9) | 24.1 (3.3) |
| Systolic blood pressure (mmHg) | 131.8 (15.4) | 132.0 (15.0) | 131.7 (15.9) | 130.7 (12.0) | 130.5 (11.8) | 130.8 (12.2) |
| Diastolic blood pressure (mmHg) | 80.3 (9.2) | 81.0 (9.3) | 79.6 (9.1) | 80.1 (7.3) | 80.4 (7.3) | 79.9 (7.3) |
| Pulse Pressure (mmHg) | NA | NA | NA | 50.6 (9.9) | 50.2 (9.6) | 51.0 (10.1) |
| Smoking | N=46,648 | N=23,993 | N=22,655 | N=34,690 | N=16,428 | N=18,262 |
| Yes (current) | 7023 (15.1%) | 4057 (16.9%) | 2966 (13.1%) | 6043 (17.4%) | 5918 (36.0%) | 125 (0.7%) |
| Past (former) | 8761 (18.8%) | 5533 (23.1%) | 3228 (14.3%) | 1331 (3.8%) | 1214 (7.4%) | 117 (0.6%) |
| No (never) | 30,864 (66.6%) | 14,403 (60.0%) | 16,461 (72.7%) | 27,316 (78.7%) | 9296  (56.6%) | 18,020 (98.7%) |
| **Glycemia** |  |  |  |  |  |  |
| FBG (mmol/l) | NA | NA | NA | 7.2 (2.4) | 7.3 (2.5) | 7.1 (2.3) |
| HbA_1C_ (mmol/mol) | 62.3 (20.7) | 62.6 (20.9) | 61.9 (20.5) | 59.8 (21.8) | 61.9 (22.6) | 57.7 (20.8) |
| **Lipid profiles** |  |  |  |  |  |  |
| Total cholesterol (mmol/l) | 4.8 (1.1) | 4.8 (1.2) | 4.9 (1.1) | 4.9 (1.1) | 4.8 (1.1) | 5.1 (1.1) |
| Triglyceride (mmol/l)^a^ | 1.7 (1.2-2.4) | 1.7 (1.2-2.6) | 1.6 (1.2-2.3) | 1.5 (1.1-2.2) | 1.5 (1.1-2.2) | 1.5 (1.1-2.2) |
| Total cholesterol: HDL ratio | 4.2 (1.3) | 4.4 (1.4) | 4 (1.2) | 3.9 (1.2) | 4.0 (1.3) | 3.9 (1.2) |
| HDL cholesterol (mmol/l) | 1.2 (0.3) | 1.1 (0.3) | 1.3 (0.3) | 1.3 (0.4) | 1.3 (0.4) | 1.3 (0.4) |
| LDL cholesterol (mmol/l) | 2.7 (0.9) | 2.7 (0.9) | 2.8 (0.9) | 2.8 (0.9) | 2.7 (0.8) | 2.8 (0.9) |
| Non-HDL cholesterol (mmol/l) | 3.6 (1.1) | 3.6 (1.1) | 3.6 (1.1) | 3.6 (1.2) | 3.5 (1.2) | 3.7 (1.1) |
| **Renal function** |  |  |  |  |  |  |
| eGFR (ml/min/1.73m^2^) | 89.6 (17.5) | 89.4 (16.8) | 89.8 (18.1) | 95.6 (17.2) | 96.3 (17.3) | 94.9 (17.1) |
| Urinary albumin | NA | NA | NA | N=34,481 | N=15,987 | N=18,494 |
| Normal |  |  |  | 27,630 (80.1%) | 12,332 (77.1%) | 15,298 (82.7%) |
| Mild |  |  |  | 5575 (16.2%) | 2986 (18.7%) | 2589 (14.0%) |
| Moderate |  |  |  | 994 (2.9%) | 517 (3.2%) | 477 (2.6%) |
| Severe |  |  |  | 282 (0.8%) | 152 (1.0%) | 130 (0.7%) |
| ACR (mg/mmol)^a^ | 1.4 (1.0-4.7) | 1.3 (1.0-5.1) | 1.4 (1.0-4.3) | 2.0 (0.7-6.0) | 1.9 (0.7-6.0) | 2.1 (0.7-6.0) |
| **Medical history** |  |  |  |  |  |  |
| Duration of diabetes | 5.0 (5.5) | 4.9 (5.3) | 5.3 (5.7) | 1.7 (3.5) | 1.5 (3.3) | 1.9 (3.7) |
| Atrial fibrillation | 717 (1.5%) | 265 (1.2%) | 452 (1.9%) | 22 (0.05%) | 14 (0.06%) | 8 (0.03%) |
| **Medications** |  |  |  |  |  |  |
| Insulin | 2947 (6.3%) | 1373 (5.7%) | 1574 (7.0%) | 2314 (5.0%) | 1318 (5.7%) | 996 (4.3%) |
| Oral hypoglycaemic agents | 31,145 (66.8%) | 15,830 (66.0%) | 15,315 (67.6%) | 25,967 (55.8%) | 13,195 (56.8%) | 12,772 (54.7%) |
| BP lowering medications | 27,465 (58.9%) | 13,760 (57.4%) | 13,705 (60.5%) | 24,798 (53.3%) | 11,971 (51.6%) | 12,827 (55.0%) |
| Lipid lowering medications | 25,321 (54.3%) | 13,386 (55.8%) | 11,935 (52.7%) | 8806  (18.9%) | 4213  (18.1%) | 4593  (19.7%) |
| Statin | 23,109 (49.5%) | 12,203 (50.9%) | 10,906 (48.1%)  Continuous data are presented as mean and standard deviation [mean (SD)] unless otherwise stated, and categorical data are presented as number and percentage [n (%)].  ^a^ presented as median and interquartile range (IQR) [median (IQR)].  Abbreviations: NA: not available; FBG: fasting blood glucose; HbA_1c_: haemoglobin A1C; HDL: high density lipoprotein; LDL: low density lipoprotein; eGFR: estimated glomerular filtration rate; ACR: albumin to creatinine ratio; BP: blood pressure. | 8126  (17.5%) | 3826  (16.5%) | 4300  (18.4%) |
